# *SUNBIM 4.0* software: new developments in small- and wide-angle X-ray scattering data analysis for scanning mode and grazing-incidence geometry

**DOI:** 10.1107/S1600576725005904

**Published:** 2025-08-13

**Authors:** Francesco Scattarella, Davide Altamura, Teresa Sibillano, Liberato De Caro, Dritan Siliqi, Cinzia Giannini

**Affiliations:** a Istituto di Cristallografia Consiglio Nazionale delle Ricerche (IC-CNR) via Amendola 122/O 70126 Bari Italy; Universität Duisburg-Essen, Germany

**Keywords:** small- and wide-angle X-ray scattering, grazing-incidence small- and wide-angle X-ray scattering, SAXS/WAXS, GISAXS/GIWAXS, computer programs, imaging, microscopy

## Abstract

*SUNBIM* (supramolecular and submolecular nano- and biomaterials X-ray imaging) is a computer suite of integrated programs which, through a user-friendly graphical interface, is able to perform a number of functions for (grazing-incidence) small- and wide-angle X-ray scattering [(GI)SAXS and (GI)WAXS] data analysis. The software combines in the same package both originally developed algorithms and reliable methods documented in the literature. Recently, new tools have been added to the original program that are particularly devoted to data reduction. The release presented in this work, named *SUNBIM 4.0*, features a new interface to perform deeper data reduction for multi-scan SAXS/WAXS, including dark-current subtraction, background evaluation and subtraction, and normalization of scattering intensity against local sample thickness derived from absorption contrast maps. In *SUNBIM 4.0*, these functionalities have been made accessible for data sets in .edf, a data format commonly used by many next-generation X-ray detectors, thanks to a new guided procedure for converting entire multi-scan data sets into a format readable by the software. A new functionality in the single data analysis section has been implemented, consisting of a semi-automatic background subtraction from the 1D profile of the azimuthal integration, corrected for a flat-panel detector geometry, to enhance peak visibility at large scattering angles (WAXS/GIWAXS). This new *SUNBIM* release will include a Microsoft Windows installer, as in the previous version, and will also be available for Mac OSX. We are confident that the new features will enable a more accurate and comprehensive analysis of (GI)SAXS/(GI)WAXS data, addressing the issues and limitations of the previous release while also enhancing the extraction of structural information contained within the data.

## Abstract 2

This work describes recent updates in the *SUNBIM* software Version 4.0, particularly for the reduction of small- and wide-angle X-ray scattering microscopy data acquired in scanning mode. The paper also presents case studies where the new routines have been applied.

## 1. Introduction

Small- and wide-angle X-ray scattering (SAXS and WAXS), and their corresponding grazing-incidence (GI) variants, are powerful techniques widely used for structural analysis in various scientific fields, including materials science, biology and chemistry (Guinier & Fournet, 1955 ▸; Glatter & Kratky, 1982 ▸; Müller-Buschbaum, 2003 ▸; Hexemer & Müller-Buschbaum, 2015 ▸). These techniques provide crucial insights into the size, shape and internal structure of nanoscale materials, as well as their crystalline properties.

However, the complexity of X-ray diffraction data requires the use of specialized software for accurate analysis and interpretation. Over the years, a variety of software tools have been developed, each offering distinct features and algorithms tailored for specific types of analysis, including calibration, azimuthal integration, indexing and 2D reconstruction of a scattering map across a sample area. Notable among these are *SAXSutilities*, a software package for on-line processing and analysis of SAXS data (Sztucki, 2022 ▸), *pyFAI*, a Python library for high-performance azimuthal integration and calibration of data from area detectors (Ashiotis *et al.*, 2015 ▸; Kieffer *et al.*, 2020 ▸), and *DAWN*, an open-source program for the visualization and processing of powder X-ray diffraction and SAXS data (Basham *et al.*, 2015 ▸; Filik *et al.*, 2017 ▸). There are also specialized software programs for more advanced analysis of SAXS patterns, focusing on fitting and modelling. Among many others, notable are *ATSAS*, a comprehensive and advanced software suite designed for analysing small-angle scattering data from biological macromolecules (Manalastas-Cantos *et al.*, 2021 ▸; Franke *et al.*, 2025 ▸), *ScÅtter*, a Java-based graphical user interface for the processing and analysis of SAXS data (Tully *et al.*, 2021 ▸), *SASview*, a sophisticated model-fitting program built around C++/Python and utilizing NIST-developed model functions (https://www.sasview.org/), and *US-SOMO*, a comprehensive open-source suite of com­puter programs centred on hydrodynamic modelling and SAXS data analysis and simulation (Brookes & Rocco, 2018 ▸). These tools have significantly advanced the field by automating complex data processing workflows and enhancing the accuracy of structural interpretations.

The *SUNBIM* software, first introduced by Siliqi *et al.* (2016 ▸), has emerged as a versatile and user-friendly platform tailored specifically for (GI)SAXS/(GI)WAXS data analysis and visualization. It offers a comprehensive suite of tools that support not only standard data processing tasks but also advanced features. The software comprises five main modules and a user’s manual section [see Fig. 1 of Siliqi *et al.* (2016 ▸)]:

(i) *Calibration*: allows calibration of (GI)SAXS/(GI)WAXS data, essential for accurate structural analysis.

(ii) *Single-scan SAXS and WAXS Data Analysis (S-SAWANA)*: dedicated to the analysis of single scans, both (GI)SAXS and (GI)WAXS, enabling 2D-to-1D data transformation (folding) and enhancing raw data quality through denoising and beam divergence deconvolution.

(iii) *Batch Script*: allows users to prepare batch scripts for sequential acquisition of 2D SAXS/WAXS frames in scanning mode, enabling efficient data collection and processing.

(iv) *Multi-scan SAXS and WAXS Data Analysis (M-SAWANA)*: manages the analysis of large data sets from multiple scans, converting thousands of 2D SAXS frames into quantitative microscopy images using advanced algorithms like multi-modal imaging and canonical correlation analysis (CCA).

(v) *One-D Data Analysis Manager*: provides tools for managing and exporting 1D profiles from 2D data, enabling further specialized analysis.

(vi) *Help*: direct link to the user’s manual.

**Figure 1:**
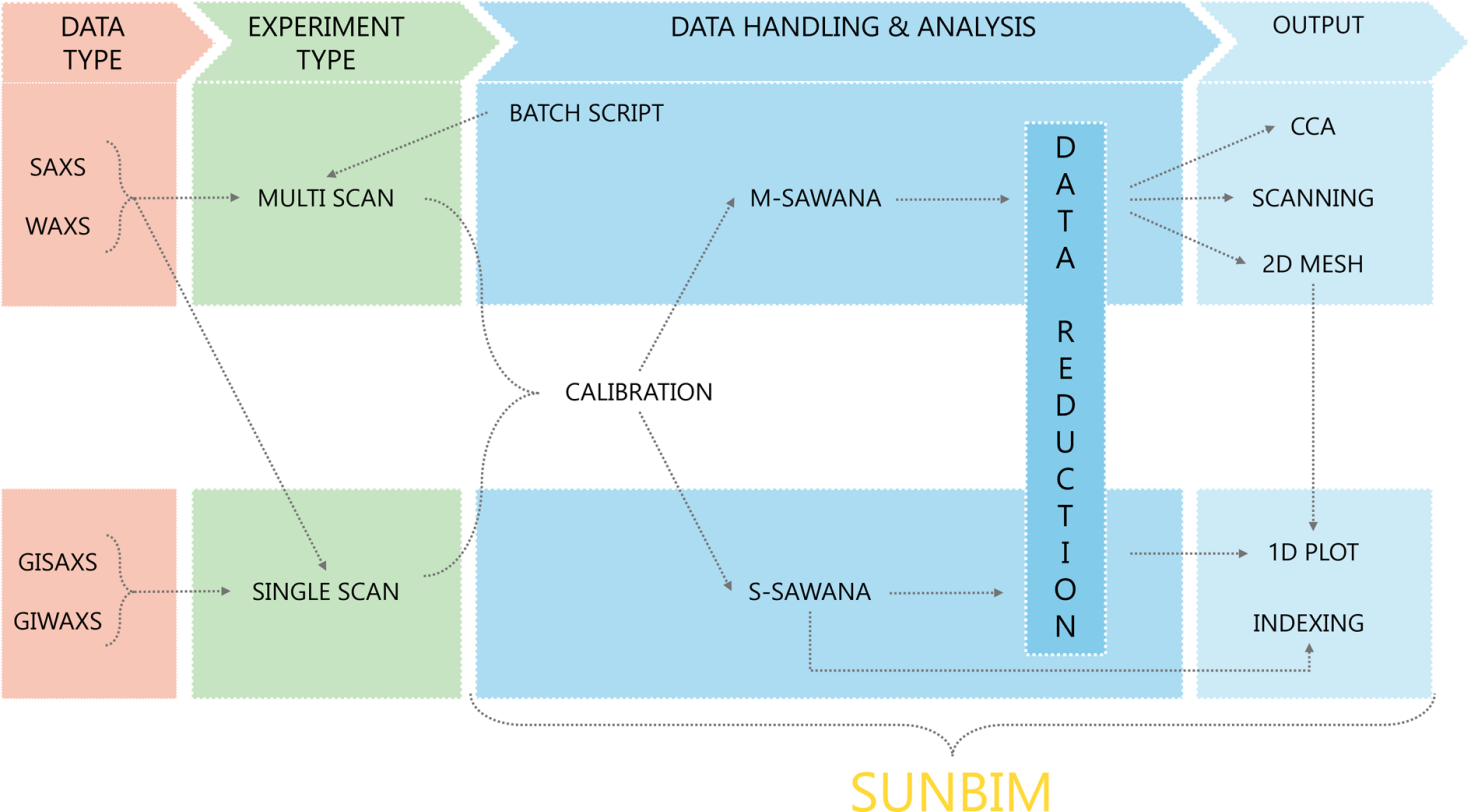
*SUNBIM*: schematic representation of data handling and analysis and their interconnections.

*SUNBIM*’s user-friendly graphical interface streamlines the data analysis process, integrating both original algorithms (*e.g.* denoising, beam centring) and well established methods from the literature [*e.g.* multi-modal imaging, (GI)SAXS indexing]. This combination allows for the preparation of X-ray dif­frac­tion data before conducting specific analyses, ensuring high-quality data processing and enabling researchers to extract quantitative information efficiently from large data sets. Fig. 1 ▸ shows the typical scheme for (GI)SAXS/(GI)WAXS data handling and analysis using *SUNBIM*. It starts with the type of data collected (or to be collected) and the experiment type (whether single or multiple scans). In the case of multiple scans, the *Batch Script* feature can be used to create a script with instructions to send to the experimental setup and proceed with data collection in a well defined area of the sample. The script is currently formatted to communicate with a Rigaku setup made up of an Fr-E+ SuperBright rotating copper anode (Cu *K*α) microsource coupled to an SMAX3000 camera [see Altamura *et al.* (2012 ▸) for more details], but it is adaptable to other configurations upon request. The *Calibration* procedure is common to both types of experiment and must be performed before proceeding with any data analysis. Following the two distinct pipelines, one reaches the *Data Reduction* section, which contains a series of features and algorithms to prepare raw data for further analysis. Depending on the type of input data, *SUNBIM* offers a range of output options. *M-SAWANA* provides:

(i) Correlation maps based on CCA, a statistical method introduced to evaluate the relationship between two sets of variables (Hotelling, 1936 ▸), used, in this context, to avoid analysing the entire data set by identifying correlations between hundreds or thousands of frames in a composite map and a few selected and meaningful profiles (Siliqi *et al.*, 2016 ▸).

(ii) Scanning colour maps (‘microscopies’) encoding the local presence or absence of a specific tissue component, the orientation of a specific direction in the tissue, and the degree of orientation.

(iii) Mesh images of transmitted and scattered intensities.

The user can interactively explore the composite image to select and visualize a specific 1D profile along with the corresponding 2D frame, or locate its position within the mesh. Once identified, the frame can be imported into *S-SAWANA* for in-depth analysis. As mentioned before, this section allows the user to load and visualize a single 2D frame and is particularly significant for those users interested in (GI)SAXS and/or (GI)WAXS data reduction. The output of *S-SAWANA* includes:

(i) One-dimensional profiles obtained through azimuthal integration over user-defined angular sectors.

(ii) One or more vertical, horizontal or radial cross sections extracted from a GISAXS image and exported into the *IsGISAXS* program format (Lazzari, 2002 ▸).

(iii) A graphical representation of the 2D (GI)SAXS frame, where the Bragg peak positions and the corresponding Miller indices are displayed (Tate *et al.*, 2006 ▸).

More details on these functionalities are given by Siliqi *et al.* (2016 ▸).

The described procedure has been successfully employed in a variety of research studies, and to date *SUNBIM* has been cited in almost 40 papers, underscoring its reliability and effectiveness in X-ray diffraction data analysis. For instance, the *M-SAWANA* package was extensively used by Camposeo *et al.* (2016 ▸) and Zhang *et al.* (2022 ▸), enabling detailed analysis in the field of materials science; by Scattarella *et al.* (2021 ▸) for the study of gel-based biocomposites; by Altamura *et al.* (2016 ▸) and Sibillano *et al.* (2016 ▸) for the study of new materials applied to regenerative medicine; by Montes-de Oca-Ávalos *et al.* (2018 ▸, 2020 ▸) and Rodríguez Pineda *et al.* (2025 ▸) for food packaging research; and by D’Amico *et al.* (2023 ▸) in the pharmaceutical field. For a single-scan analysis, the *S-SAWANA* package has been employed on WAXS data in the study of peptide-based fibres (Diaferia *et al.*, 2018 ▸; Rizzo *et al.*, 2022 ▸) and collagen-based tissues (Terzi *et al.*, 2018 ▸), as well as on GISAXS data for the investigation of perovskites (Toso *et al.*, 2021 ▸). Some *SUNBIM* routines have also been used to process WAXS scan data of regenerated cellulose fibres at mesoscopic resolution (Johansson *et al.*, 2024 ▸).

Nevertheless, the earlier version of *SUNBIM* (Siliqi *et al.*, 2016 ▸) had some limitations. In its subsequent upgrade (Scattarella *et al.*, 2020 ▸) the normalization to mass thickness distribution was implemented and applied to SAXS data, then successfully applied to Bragg diffraction data as well (D’Amico *et al.*, 2023 ▸). However, the 2020 upgrade was unable to reconstruct 2D maps from SAXS/WAXS scans saved in the .edf file format, which is widely used by many leading X-ray detectors, such as PILATUS and Eiger by DECTRIS. This limitation restricted the software’s utility for users working with these popular detectors. Additionally, the software offered a limited range of tools for data reduction of 2D maps and 1D profiles, which constrained the depth of analysis that could be performed within the software. Furthermore, that version of *SUNBIM* was only available for the Microsoft Windows operating system, limiting its accessibility to researchers using other platforms (such as Mac OSX).

The present study introduces the new *SUNBIM 4.0* upgrade, which includes a thorough revision of many features from previous versions and introduces new ones. Section 2 will describe the new .edf image upload function and the new data reduction window developed in the *M-SAWANA* section. Examples of data reduction applied to scans of samples of polymeric beads loaded with budesonide will also be shown. Section 3 will describe the new background subtraction method for (GI)WAXS patterns implemented in *S-SAWANA*, with examples of its application on antibacterial nano­structured surface patterns. Finally, the supported platforms and distribution will be indicated in Section 4.

These updates aim to simplify the data analysis process further, reduce computational time and increase the accuracy of structure determinations. By addressing the evolving needs of the research community, the latest version of *SUNBIM* promises to strengthen the analytical capabilities available to scientists utilizing (GI)SAXS/(GI)WAXS techniques for materials characterization.

## 2. Multi-scan SAXS and WAXS data analysis (*M-SAWANA*)

### 2.1. New conversion procedure

**Figure 2:**
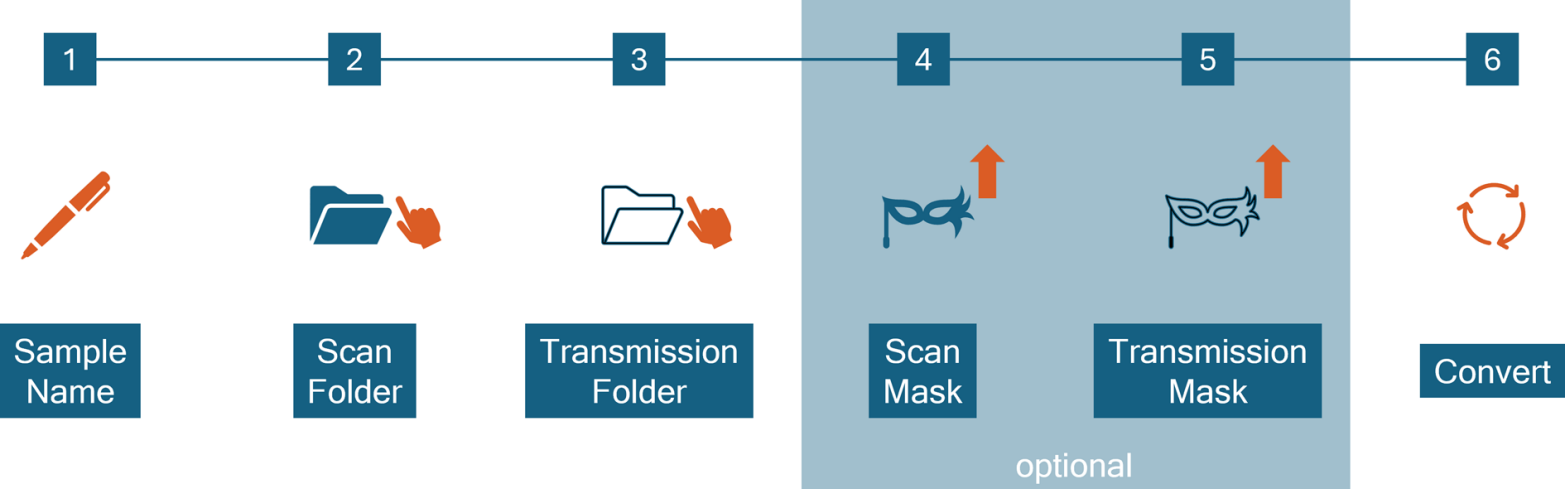
Schematic representation of the .edf data conversion procedure. As indicated in the figure, the upload of binary masks for scattering and transmission data is optional.

As in the previous version, *SUNBIM 4.0* provides the possibility of managing files in different formats, such as .tiff, .cbf, .edf, .ccd, .mpa and .mat for 2D images, and .dat for 1D profiles and batch script (ASCII files). However, many of them could not be used in the section dedicated to the composition and visualization of scanning microscopies. As described by Siliqi *et al.* (2016 ▸), following SAXS or WAXS data collection across a mesh area, the gathered 2D frames can be merged into a single composite image. Similarly, a unified mesh image of transmitted or scattered intensities across the entire scattering angle range can be created. This composite image allows interactive selection and plotting of specific 1D profiles, aiding in data exploration. However, in the previous version these features were limited to data collected in a specific proprietary format of Rigaku (.mpa), which associates each collected frame with a text file (.info) containing a series of information regarding the collected data (such as scan position, exposure time, transmitted beam intensity). In *SUNBIM 4.0*, a new menu has been added that allows for importing of files in .edf format and making them ready for the composite function after an automatic conversion procedure. An important option has been added to this conversion procedure: the ability both to extract all the necessary information present in the metadata saved within the .edf file and to associate, if available, the information related to the transmitted signal. For example, if during a data acquisition session both scattering and transmission data were collected on the sample (even if not simultaneously), these data can be associated and used for generating composites and individual scattering and transmission maps. Furthermore, during the conversion stage, it is possible to load one or two binary masks (one for scattering and one for transmission data) aimed at excluding any defective pixels or empty areas of the detector. A schematic representation of the described steps is shown in Fig. 2 ▸. The first and very recent example of application of the new *SUNBIM* release to SAXS/WAXS microscopies obtained from .edf files can be found in the report by Rodríguez Pineda *et al.* (2025 ▸).

### 2.2. Data reduction

As already mentioned, one of the most interesting features of *SUNBIM* is the ability to create an interactive map of the scanned area of a sample. Montes-de Oca-Ávalos *et al.* (2020 ▸) and Scattarella *et al.* (2021 ▸) both used this feature extensively for multiscale sample analysis. By reconstructing a composite map of SAXS scans performed on various samples, specific regions of interest (*e.g.* areas with anomalous scattering signals) were identified. Subsequently, after examining the 1D profiles in those areas, WAXS acquisition was carried out in the selected coordinates, enabling fast and straightforward multiscale sample characterization by comparing SAXS and WAXS signals. In this section, we describe the main new features related to data reduction introduced in *SUNBIM 4.0*, such as:

(i) dark-frame subtraction;

(i) transmission coefficient and background estimation; and

(iii) relative thickness evaluation.

The first two procedures are well established in the literature and are commonly used in SAXS/WAXS data processing workflows. To the best of our knowledge, the third feature, the evaluation of relative thickness, represents an original contribution by our software and is not currently available in comparable tools.

#### 2.2.1. Dark-frame subtraction

Dark-frame correction is now possible for scattering map calculation, involving the subtraction of a dark-frame image acquired with the beam off, which can be loaded and used as a reference. The purpose is to correct the main components of the detector artefacts, including bias current, dark current and readout noise. If the transmitted beam intensity is measured by the detector, as in the XMI-Lab setup described by Altamura *et al.* (2012 ▸), users can also input the measured value of the dark current for that instrument. Note that dark-current signals should be obtained for integration times sufficient to achieve a signal statistically comparable to that of the raw data, to avoid noise increase during subtraction. To ensure that the dark-current signal values are compatible with the experimental ones, the program automatically normalizes the loaded values to the actual integration time.

#### 2.2.2. Transmission coefficient and background estimation

Proper data reduction involves identifying the background signal from the experimental data. This is important for two reasons: (i) it allows for the calculation of transmission maps and (ii) it enables the removal of the instrumental contribution from the measured scattering signal. This signal may or may not be contained within the scanning area (*e.g.* vacuum or substrate area). In the former case, the background signal can be identified in an area where the transmission coefficient is a maximum. In the latter case, it can be obtained from a different data set. In this context, the user has a variety of options to produce customized data handling with the new *Data Reduction* panel. First, the local transmission coefficient *T*(*x*, *y*) can be calculated in different modalities, depending on the experimental conditions. *T*(*x*, *y*) is defined as
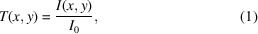
where *I*_0_ is the incoming X-ray beam intensity and *I*(*x*, *y*) is the output intensity after the interaction with the sample in the scan position (*x*, *y*). So, if the scanning area contains a no-sample region, *I*_0_ can be assumed to be equal to:

(i) the maximum value 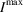 of the transmitted beam intensity;

(ii) the maximum value 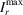 for each line *r*; or

(iii) the average value *I*^ave^ of the transmitted beam intensity over a background region identified by the user.

**Figure 3:**
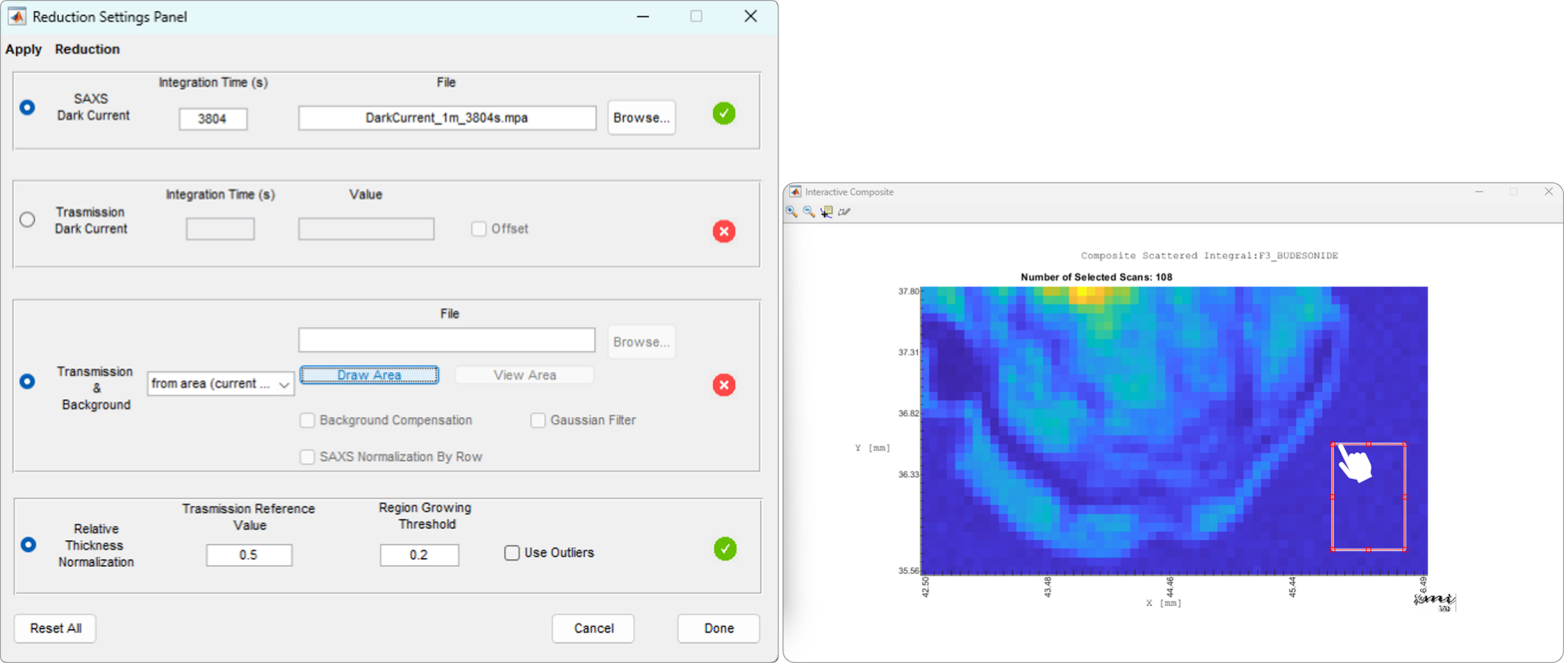
The new *Data Reduction* panel. On the left is a screenshot of the menu, and on the right is an example of how to select the background area manually from the composite map of a scan conducted at the XMI-Lab on polymeric microspheres containing budesonide (D’Amico *et al.*, 2023 ▸).

If the scanning area does not contain a background region, the user can load an external file (*e.g.* from another data set) which can contain a single transmitted intensity value or an average value detected over a specific region. The program assumes that transmitted intensity as the current *I*_0_ and calculates both the local transmission coefficient *T*(*x*, *y*) and the background signal to be subtracted from the raw SAXS/WAXS scattering signal to obtain the effective scattering map (see Section 2.3 for details). Finally, in order to make the data reduction as customized as possible, this panel allows for the introduction of additional options to be applied to the thus-obtained transmission image, as shown in Fig. 3 ▸ (left-hand panel), such as the application of a Gaussian filter and an *Irregular Background Compensation* algorithm (Scattarella *et al.*, 2017 ▸). Also in Fig. 3 ▸, in the right-hand panel, an example of background area selection is shown on a 2D map, acquired at the XMI-Lab, of a polymeric bead containing budesonide (D’Amico *et al.*, 2023 ▸).

#### 2.2.3. Relative thickness

*T* maps generally depict lateral variations in the product of the X-ray absorption coefficient μ(*x*, *y*) (*i.e.* of the local material density) and local thickness *t*(*x*, *y*) of the sample. Assuming a reference sample with a constant thickness *t*_ref_, *t*(*x*, *y*) can be expressed as a multiple *n*(*x*, *y*) of *t*_ref_ such that
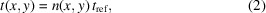
with *n*(*x*, *y*) > 0. In the case of approximately constant and equal absorption coefficients for both uneven and reference samples [μ(*x*, *y*) ≃ μ_ref_(*x*, *y*) ≃ 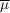], the Lambert–Beer law (Als-Nielsen & McMorrow, 2011 ▸) allows the following expression: 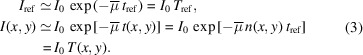
Here, *I*_ref_ and *T*_ref_ are the average transmitted signal and the average transmission coefficient of the reference material, respectively, and 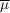 is the average absorption coefficient, equal for both the sample and the reference value. From equation (3), *T*(*x*, *y*) can be rewritten as
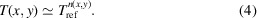
In this way, from the measured transmission map *T*(*x*, *y*), it is possible to estimate the map of relative thickness *n*(*x*, *y*) according to the following equation: 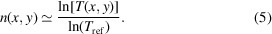
So, by defining the value of the reference mean transmission coefficient *T*_ref_, it is feasible to represent a map of the sample relative thickness *n*(*x*, *y*) and subsequently utilize it to normalize the measured scattering intensity to obtain a scattering map independent of thickness variations (see next section). Moreover, according to equation (5), the *n*(*x*, *y*) map will highlight the thickness variations that are responsible for the observed differences in the transmission map. In practice, in the *Data Reduction* panel the user can choose to compute the map of relative thickness by setting a reference value of the transmission coefficient (*e.g. T*_ref_ = 0.5) and, if necessary, use the obtained result to normalize the scattering maps. In the case of scanning areas containing void or non-sample regions, the calculated relative thickness would be approximately zero. Therefore, in order to avoid singularities arising from the normalization to arbitrary low *n* values in those regions, it is possible to use a masking procedure based on the *Region Growing* algorithm (Pratt, 2007 ▸), as done by Scattarella *et al.* (2021 ▸), to exclude non-sample areas.

### 2.3. Composite and visualization

One of the major innovations in *SUNBIM 4.0* is the complete reorganization of the *Composite and Visualization* section in *M-SAWANA*, together with the new functionalities of the aforementioned *Data Reduction* panel.

#### 2.3.1. Composites

Primarily, besides the composition of the as-collected 2D SAXS frames into a single image (mesh), there is a new capability to generate composite 2D maps of scattering and transmission signals integrated over the entire scattering angle range. These maps are calculated from, respectively, the 2D SAXS/WAXS frames and the transmission signals collected during scanning. In addition, two more maps have been included, one displaying the SAXS/WAXS signal normalized to the transmission coefficient described in Section 2.2.2, and the other showing the relative thickness as described in Section 2.2.3. Users can interactively choose which of the four resulting maps to use during analysis. The advantage of directly manipulating integrated signal maps lies primarily in the ability to identify more easily and reliably areas of interest within the sample and to distinguish signals of pure scattering from those resulting from increased absorption and/or sample thickness.

#### 2.3.2. Map visualization

This section of *M-SAWANA* provides the capability to visualize false-colour images of mesh-like scattering and transmission maps. Unlike in the previous version, in *SUNBIM 4.0* it is also possible to generate effective scattering maps, which account for both background signal and sample thickness variations. Firstly, on the basis of the settings configured in the *Data Reduction* menu (refer to the previous section for details), users can identify any background areas, if present in the scan, and utilize the estimated signal to subtract it from the measured signal within the sample. The scattering intensity can then be normalized to the transmission coefficient maps (as for the composite) in order to provide a ready correction of the scattered intensity according to the local absorption coefficient variations, as required in any SAXS/WAXS experiment. So the normalized scattering thus obtained can, in principle, highlight the differences in scattering contrast. As explained by Montes-de Oca-Ávalos *et al.* (2020 ▸), in the case of constant film thickness the transmission maps would directly provide the lateral variation in the X-ray absorption coefficient, *i.e.* in the local material density, whereas if the material density can be assumed constant, the map will directly provide thickness variations. These differences can be further amplified by normalizing the thus-calculated intensity to the sample thickness, resulting in an image that unequivocally indicates regions of pure scattering, *i.e.* scattering per unit mass, as demonstrated by Scattarella *et al.* (2021 ▸). From a computational point of view, with (*x*, *y*) being the scan position, the map depicting effective scattering 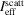 can be expressed as 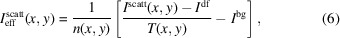
where *I*^scatt^ is the measured scattering intensity integrated over the whole *q* range [*q* = (4π/λ) sin θ, where θ is half the scattering angle and λ is the wavelength of the incident radiation], *I*^df^ is the dark-frame intensity, and *T* and *n* are the transmission coefficient and the relative sample thickness, respectively, measured at the corresponding scan position. *I*^bg^ is the scattering background intensity and can be obtained in different modalities, depending on the options set in the *Data Reduction* panel (see previous section). For example, if a background region outside the sample has been acquired during data collection, *I*^bg^ can be calculated by averaging the scattering intensity over *M* frames in that region:
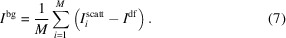
Therefore, plotting the scattered intensity normalized to the *T* coefficient and to the relative thickness *n* results in a spatial map representing the effective scattered intensity as if every point on any of the samples experienced the same absorption and had the same thickness. Such a description relies on the assumption of homogeneous sample density, so that the scattering signal is directly proportional to the sample volume. Its validity can be verified *a posteriori* on the normalized SAXS/WAXS microscopies for each specific case study. For a constant-density material, normalization to *n* will lead to totally flat (*i.e.* no) contrast. On the other hand, any residual contrast in the normalized microscopies will reveal a density/structural change. The importance of normalization to thickness variations has been proved by Scattarella *et al.* (2021 ▸) and D’Amico *et al.* (2023 ▸), where a generalization of SAXS data analysis from nano-vesicles in bulk gels of arbitrary shape and the study of concentration variations of crystalline drugs in polymeric microspheres were reported, respectively, as described in the following section. Equation (6) is also used to calculate the 1D profile of the SAXS/WAXS data following the *azimuthal integration* [see Siliqi *et al.* (2016 ▸)]. In this manner, in the *Analysis* section of *M-SAWANA* it will be possible to visualize interactively both the plot of the raw scattering data *I*^scatt^ and the effective one 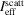: the former will provide values normalized to the average value of the whole map, to highlight variations from the average across a given sample area, while the latter will provide the actual intensity values to allow for comparison among different samples. Also in the *Analysis* section it will be possible to reconstruct the effective scattering maps within a specific *q* range, as already demonstrated in previous reports (Scattarella *et al.*, 2021 ▸; D’Amico *et al.*, 2023 ▸).

#### 2.3.3. Case study

**Figure 4:**
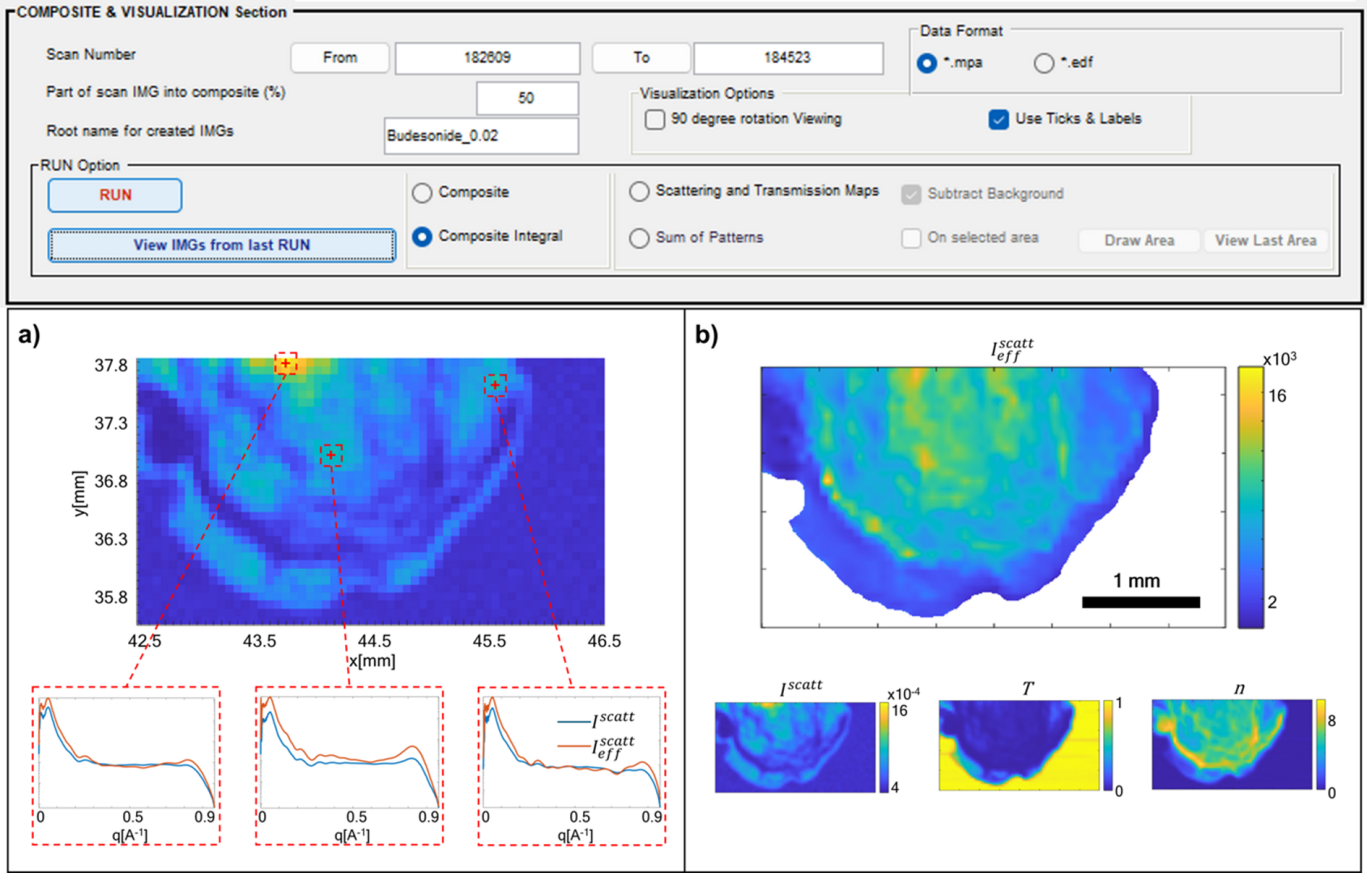
(Top) The new *Composite and Visualization* section. (*a*) The composite of a SAXS scan acquired at the XMI-Lab on a polymeric bead loaded with budesonide (D’Amico *et al.*, 2023 ▸). The thus-obtained composite can be used to extract 1D profiles interactively from the selected scans (indicated by red crosses). The plots at the bottom show a comparison between *I*^scatt^ (blue) and 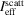 (red) profiles obtained before and after data reduction. In both cases, a denoising algorithm has been applied (Siliqi *et al.*, 2016 ▸). (*b*) Maps generated from the composite based on the parameters set in the *Data Reduction* panel: at the top the 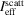 map calculated according to equation (6), and at the bottom the *I*^scatt^, *T* and *n* maps.

In Fig. 4 ▸, the top panel displays the new *Composite and Visualization* section of *M-SAWANA*, and the bottom panels show representative microscopies obtained using *SUNBIM 4.0*. In Fig. 4 ▸(*a*), the composite of the aforementioned SAXS scan conducted at the XMI-Lab on microsphere samples containing budosenide (D’Amico *et al.*, 2023 ▸) is depicted, enabling the point-by-point display of the integrated scattering intensity over the entire *q* range and allowing interactive extraction of 1D profiles integrated along the azimuth. Three profiles corresponding to scans indicated by the red crosses on the composite are represented. Within the same plot, both the raw scattering intensity profile (blue line) and the effective one obtained after data reduction (red line) are depicted. For better data readability, the profiles were processed with a denoising algorithm, available in the *One-D Data Analysis Manager* section of *SUNBIM* (Siliqi *et al.*, 2016 ▸). This is because the SAXS data set is highly noisy, due to the strong absorption of the sample, as evident in the transmission coefficient *T* map at the bottom of Fig. 4 ▸(*b*); the *I*^scatt^ and relative thickness *n* maps are also displayed. In the main part of Fig. 4 ▸(*b*), the 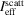 map is shown, representing the scattering signal normalized to the transmission coefficient and relative thickness point by point. Therefore, assuming a uniform material density, this is equivalent to a scattering map per unit volume, so that any residual contrast can be attributed to structural and/or local density variations.

#### 2.3.4. Averaged pattern

Finally, the new version of *M-SAWANA* can also calculate the SAXS/WAXS diffraction pattern averaged over the entire scanning area, or over a partial area selected by the user with the option *Sum of Patterns* (see top panel in Fig. 4 ▸). This approach enables the derivation of different diffraction patterns from various sample regions with a better signal-to-noise ratio and background subtracted, helping a comparative analysis for a robust quantitative assessment of the acquired data.

## 3. Single-scan SAXS and WAXS data analysis (*S-SAWANA*)

### 3.1. Background correction for (GI)WAXS data

As mentioned in the *Introduction*[Sec sec1], *S-SAWANA* is the section of *SUNBIM* dedicated to the analysis of single SAXS or WAXS scans, in either transmission or reflection conditions (GISAXS/GIWAXS). The background subtraction procedure was first developed for data sets acquired with the setup described by Altamura *et al.* (2012 ▸), where a Fuji image-plate detector and RAXIA off-line scanner were used to collect (GI)WAXS data. In that case, the background is mostly due to instrumental noise, which produces a more or less homogeneous signal across the entire plate. An effective method commonly used to remove the background to highlight diffraction peaks is to estimate it by interpolation of the 1D profile. Once the background line is determined, it is subtracted from the scattering profile. However, this approach carries the risk of subtracting any minor or diffuse scattering signals (amorphous) if they are incorrectly classified as background. The updated version of *S-SAWANA* has been en­riched with a new procedure to subtract the background signal from the single 2D scan after azimuthal integration, to achieve a reliable 1D profile ready for extraction of quantitative information. This procedure is crucial in contexts where the collected scattering signal is so weak that it is on the same scale as the background (*e.g.* fast scanning microscopy). The effect becomes more pronounced as the sample-to-detector distance decreases, making it highly evident under (GI)WAXS conditions, especially at high scattering angles (*e.g.* 2θ > 40°). In the new version of *S-SAWANA*, during the 2D → 1D folding phase, a spherical angle correction is automatically applied to the calibrated 2D data *I*^scatt^(*x*, *y*). Actually, in order to account for the projection of the flat detector pixels on the virtual sphere generated by the scattered wavefront, resulting in a difference in solid angle covered by each pixel (Bösecke & Diat, 1997 ▸; Pauw, 2013 ▸), the diffraction pattern 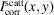 is corrected pixel by pixel by the following quantity:
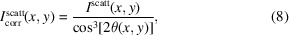
where 2θ is the scattering angle. This correction is applied both for the estimated background and for the scattering signals.

In this way, a user can evaluate background from the signal image itself, selecting an area out of the diffraction pattern and averaging the intensity over that region. The *S-SAWANA* function automatically suggests two possible background areas: one near the direct beam position (where the beam stopper is typically located) and the other where the signal can be considered nearly negligible (*i.e.* at the corners of the detector). If these options are not satisfactory, the user has the chance to select a background area by drawing it manually. Subsequently, a constant 2D background image is generated and used to subtract the background signal from the 2D pattern. As the same spherical angle correction is applied to the background image, the resulting 1D plot contains, in principle, only the correct scattering signal of the sample projected on a spherical surface. The implemented procedure allows for:

(i) enhancing the visibility of peaks at high *q* values;

(ii) optimizing the peak intensity for the potential identification of preferential crystalline domain orientation;

(iii) preserving the diffuse scattering signal (amorphous);

(iv) correcting peak positions and optimizing the whole profile for subsequent fitting procedures.

**Figure 5:**
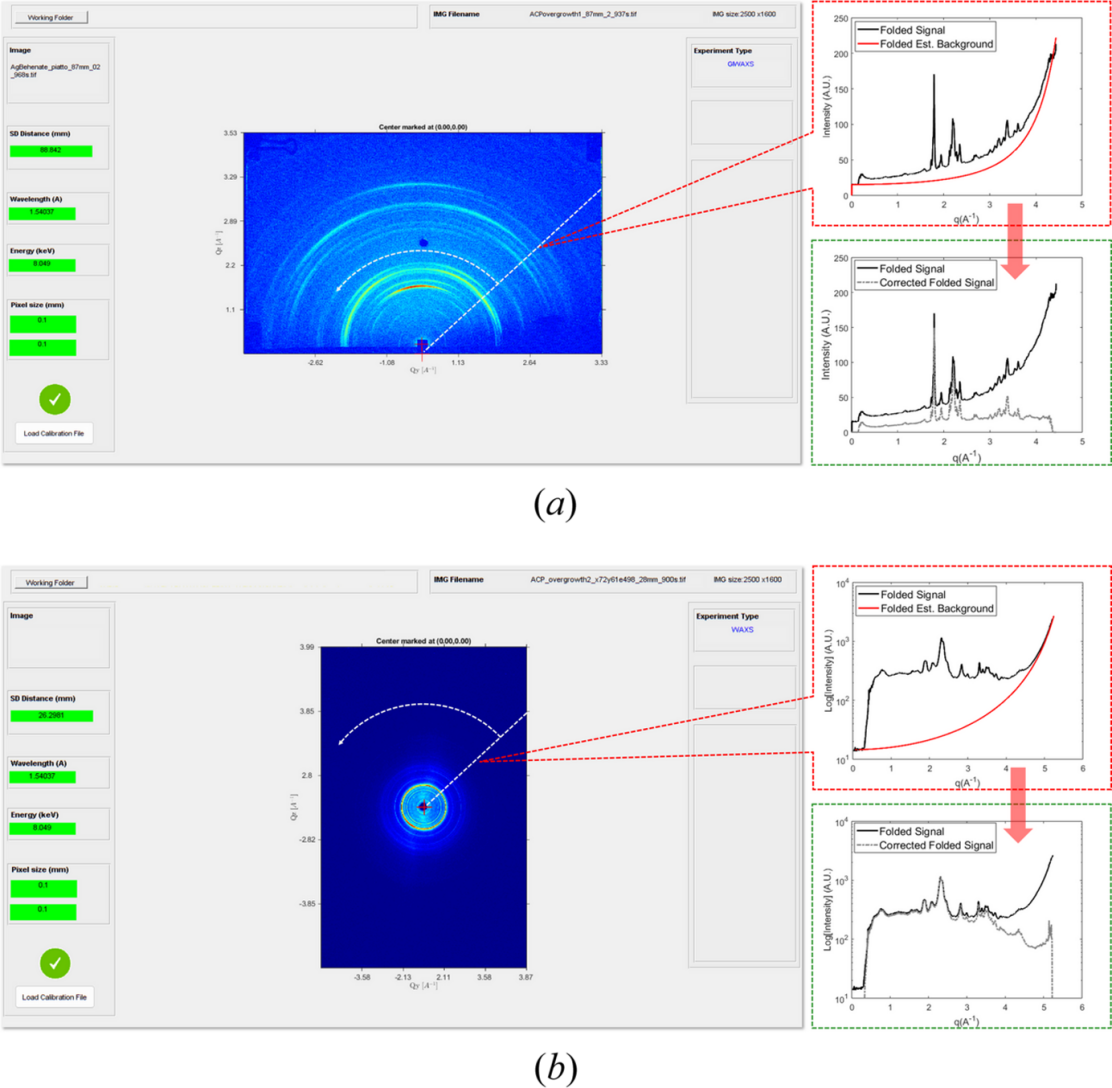
(*a*) GIWAXS and (*b*) WAXS of ANSs from ACP in a growth solution (GS) analysed using *SUNBIM 4.0* (Degli Esposti *et al.*, 2024 ▸). The panels on the left show the *S-SAWANA* window for both cases. The panels on the right display the results of the corresponding azimuthal integration (folding): at the top of each pair is the 1D profile obtained by applying the spherical angle correction (black) and the corresponding estimated background profile (red); at the bottom of each pair is the resulting profile after subtraction (grey dashed line) compared with the initial one (black). A significant improvement in the visibility of the high-*q* diffraction peaks is observed.

This function has recently been used to analyse the GIWAXS and WAXS diffraction patterns of antibacterial nanostructured surfaces (ANSs) collected at the XMI-Lab (Degli Esposti *et al.*, 2024 ▸). Here, ANSs composed of bioactive amorphous calcium phosphate (ACP) nanocrystals were analysed using various methodologies. Fig. 5 ▸ shows two examples of (*a*) GIWAXS and (*b*) WAXS patterns of ACP surfaces, acquired at distances of approximately 87 and 28 mm, respectively. On the left, the *S-SAWANA* window is displayed in both cases, while on the right, two plots are shown: the first, highlighted by a dashed red outline, shows the scattering profile after azimuthal integration (black line) and the background profile (red line) estimated in the region of the beam stopper; the second, highlighted by a dashed green outline, shows the initial scattering profile (black line) compared with the profiles after background subtraction (dashed grey line). As seen here, background subtraction becomes crucial for *q* > 4 Å^−1^ (*i.e.* 2θ > 60°), as the scattering signal is comparable to the background at these *q* values, but this situation can also occur at lower values. Therefore, for weaker signals, this procedure becomes necessary to obtain an optimal scattering signal for 1D profile fitting procedures. In fact, relevant quantitative results were obtained by fitting the whole profile of the WAXS and GIWAXS data reported here [see the supporting information linked to the report by Degli Esposti *et al.* (2024 ▸)] using the *FULLPROF* program (Rodriguez-Carvajal, 2001 ▸), demonstrating the importance of the implemented background subtraction procedure.

## 4. Supported platforms and distribution

*SUNBIM 4.0* is programmed in MATLAB and can be downloaded for free (for academic users after registration) from https://www.ba.ic.cnr.it/softwareic/sunbimweb/. It is available as a standalone executable for Microsoft Windows (64-bit versions) and Mac OSX (Apple Silicon). The distribution includes the free MATLAB Compiler Runtime Library. Dritan Siliqi and Francesco Scattarella oversee the software assembly, maintenance and future updates, with contributions from the XMI-Lab team: Davide Altamura, Teresa Sibillano, Liberato De Caro and Cinzia Giannini.

## 5. Conclusions

*SUNBIM* is a suite of integrated programs for X-ray imaging of nano- and biomaterials using SAXS, WAXS, GISAXS and GIWAXS techniques. The *SUNBIM 4.0* release introduces a set of new functionalities that are particularly devoted to data reduction and handling of the scanning X-ray microscopy data set. It includes a new interface to perform dark-current subtraction, background evaluation and subtraction, and local transmission and relative thickness normalization of scattering signals. These features, originally developed only for a single data format (.mpa), have been extended to support .edf files, a data format commonly used by SAXS/WAXS laboratory instruments. However, the authors are open to the integration of new formats based on user needs. In particular, future releases aim to include support for .h5 data encoding, which is widely used at synchrotron facilities. Moreover, for single WAXS/GIWAXS data analysis, a semi-automatic background subtraction from the 1D profile of the azimuthal integration, adjusted for the flat-panel detector geometry, has been added to improve peak visibility at higher scattering angles.

All these features have been tested on various samples and we presented in this work some experimental applications. Finally, *SUNBIM 4.0* has been made available on MacOSX platforms (in addition to Microsoft Windows). Following feedback from the user community, future releases will be available for Linux platforms.
